# Proteomic Analysis of *Arachis hypogaea* Seeds from Different Maturity Classes

**DOI:** 10.3390/plants13081111

**Published:** 2024-04-16

**Authors:** Ashley Cherry, Brian Fisher, William Branch, Christopher Peralta, Lissa Gilliam, Olga Pahom, Chris Liebold, Julie Marshall

**Affiliations:** 1 Department of Mathematics, Lubbock Christian University, Lubbock, TX 79407, USA; ashley.cherry@lcu.edu (A.C.); brian.fisher@lcu.edu (B.F.); 2 Institute of Plant Breeding, Genetics, and Genomics, Tifton Campus, The University of Georgia, Crop and Soil Sciences, Tifton, GA 31793, USA; wdbranch@uga.edu; 3 The Proteomics Resource Center, Rockefeller University, New York, NY 10065, USA; cperalta@rockefeller.edu; 4 Biochemistry Research Laboratory, Lubbock Christian University, Lubbock, TX 79407, USA; lissa.gilliam@lcu.edu; 5 Honors College, Lubbock Christian University, Lubbock, TX 79407, USA; olga.pahom@lcu.edu; 6 The J. M. Smucker Co., Orrville, OH 44667, USA; chris.liebold@jmsmucker.com; 7 Department of Chemistry and Biochemistry, Lubbock Christian University, Lubbock, TX 79407, USA

**Keywords:** seed maturity, proteomics, peanut, desiccation tolerance

## Abstract

Physiological maturity impacts seed quality through various mechanisms including vigor, desiccation tolerance, dormancy induction, synthesis of raw materials (including seed storage proteins), and the reorganization of metabolisms. Peanut seed development can be classified into seven classes with four incremental stages per class. Based on the mesocarp color, the final three stages are commonly referred to as “orange”, “brown”, and “black”. In 2017, freshly harvested pods from one genotype of runner market-type peanuts grown under conventional practices were obtained from the University of Georgia research facility. The pods were removed from the plant material and ‘pod blasted’ to reveal the mesocarp. After separation, the remainder of the pod outer layer was removed, and the seeds were segregated for proteomic analysis. The raw peanuts were analyzed by bottom-up LC-MS/MS proteomics, which was conducted by the Proteomics Resource Center at the Rockefeller University, to identify the significant protein composition differences in each maturity class. The proteomic data revealed differentially expressed proteins as a function of maturity class with multiple functions including plant defense, metabolism, cell signaling, nutrient accumulation, and packaging. Understanding the processes needed for seed maturation will enable peanut scientists to evaluate the traits needed for robust germination, hardiness of the seed in response to disease, and nutrient quality.

## 1. Introduction

The cultivated peanut (*Arachis hypogaea*) seeds in this study include proteins, lipids, and starch, which provide the necessary energy for the plant’s development. The harvested seeds have a variety of uses, including biofuel production and consumption by humans and livestock [1,2,3]. Legumes such as peanuts can also fix nitrogen, which can improve sustainability in industrial agriculture [4]. As the world population continues to grow, there is an increased pressure for the peanut seed to be used as a source of plant-based protein and oilseed. To meet this growing demand, particularly in the agronomic sector, it is necessary to improve peanut seed performance.

Physiological maturity impacts seed quality through a variety of mechanisms including desiccation tolerance, preparation of storage reserves, and the establishment of dormancy [5]. Maturation is characterized as a period of reserve accumulation and the reorganization of metabolisms, as well as a synthesis of starch, storage proteins, and oil. Desiccation, which is characterized by water loss, is also an active stage in terms of gene expression and metabolism [6]. Drying the seed allows it to maintain viability for a long period of time, thereby maintaining low levels of metabolic activity and other processes that improve vigor. In 2011, Hajduch et al. [6] characterized the proteome of oilseeds to understand the accumulation of storage reserves in crops, particularly protein and oil content. Post-translational modifications (PTM) demonstrated a possible control of metabolism and organizations of storage reserves through the phosphorylation of caleosins and stereoleosins. In 2012, Li et al. [7] investigated the proteome through seed development, stating that proteomics provides information about protein abundance, protein sequence, and PTMs as a means through which to understand complex protein dynamics and regulatory mechanisms in comparison to genomic and transcriptomic approaches. Another study described the metabolic signatures of lipid accumulation in seeds using proteomic techniques from 6 developmental stages and 10 functional categories [8]. The results suggested that cellular metabolic events are controlled by protein–protein interactions, PTMs, and enzymatic activities that are not described by transcriptional approaches alone. Previous research has demonstrated the value of proteomic analysis to study seed development in oilseeds and other cropping systems.

Proteomics is a tool that has been used to understand protein expression and the regulatory mechanisms involved in the accumulation of storage reserves in oilseeds such as *Brassica campestris* L. (field mustard) and *Jatropha curcas* (a type of castor oil) [7,8]. In peanuts, proteomic analysis has been used to characterize specific genotypes, lipid accumulation and transport, fatty acid pathways, response to disease, skin composition, allergens, and pod development [9,10,11,12,13,14,15]. Previous studies have described the allergens present in mature seeds, skins, and skins from phenolic extraction, as well as relative allergenic reactivity [9,10]. Other studies have focused on lipid accumulation and dynamics during pod formation [11,12]. In 2013, Zhao et al. [13] reported on their transcriptomic and proteomic analysis during *Aspergillus flavus* infection following artificial inoculation to better understand the defense mechanisms of the skin and the whole seed post infection. Li et al. [14] described the peanut proteome of seeds at different stages of underground development using pod and seed size to distinguish between seven developmental stages. Li’s study focused on the accumulation of seed storage proteins (SSPs) to identify allergens and align proteomic sequences to the A and B genome of peanuts. In contrast to the study described presently, the final stage of seed development reported by Li et al. [14] includes the last three stages of pod color, which are revealed by pod blasting and focusing on the critical processes that occur to prepare lipids, protein, and other components for desiccation, storage, and germination. In 2019, Zhou et al. [15] reported on the total oil, protein, and sugar content in each pod-blasted maturity class and demonstrated that total protein and lipid amounts do not change significantly during the last phase of peanut seed maturation, which suggests the need for additional investigations of these dynamic changes. The impact of seed maturity on germination efficiency and peanut seed quality is important to all segments of industry, including producers and consumers. The present study focuses on the last stage of development, in which protein and oil quantity does not change significantly, and this is achieved by examining the processes and dynamic changes occurring to the storage reserves post-accumulation.

To evaluate physiological maturation, previous research has employed a method of classification based on the color and morphological differences of the mesocarp to investigate the developmental stages of fresh peanuts [16]. Determining maturity using this method involves the elimination of a part of the esocarp to expose the pod mesocarp. The pericarp undergoes progressive darkening, which results in mesocarp colors ranging from white (immature) to black (mature). Once the outer layer of the pod is taken away, the seed maturity can be determined and classified (white, yellow, orange, brown, or black) without damaging the seeds, thus permitting further physiological and chemical investigations related to the seeds’ stage of development.

Given the findings from other plants and peanut-focused studies, proteomics presents a method for understanding the difference in protein expression and functionality when focusing on the last critical stage of seed development. The specific aim of this project is to clarify the changes in protein composition between seeds from different maturation stages. To accomplish this aim, seeds were organized into different classes (orange, brown, and black) as revealed by pod blasting, and the proteins were extracted and identified. Differences in protein content determined to be statistically significant were identified and sorted into functional classes to provide a global picture of the active processes in the maturation of the peanut seed. 

## 2. Results

In a previous related study, pooled samples from the three maturity classes, “orange”, “brown”, and “black”, were analyzed to determine macronutrient content, which averaged 50% oil and 20% protein (as expected when compared to the nutritional standards for raw peanuts [15,17,18]). Although the samples were similar in size and nutrient composition, the roasting and quality attributes were different. Immature samples do not roast as efficiently as more mature seeds, and they do not generate a robust roasted peanut flavor. Additionally, the less mature samples do not germinate as efficiently as more mature samples [15]. The inferiority of immature sample performance in the finished product prompted a further investigation of the composition and abundance of seed storage proteins in each maturity class. 

**Figure 1:**
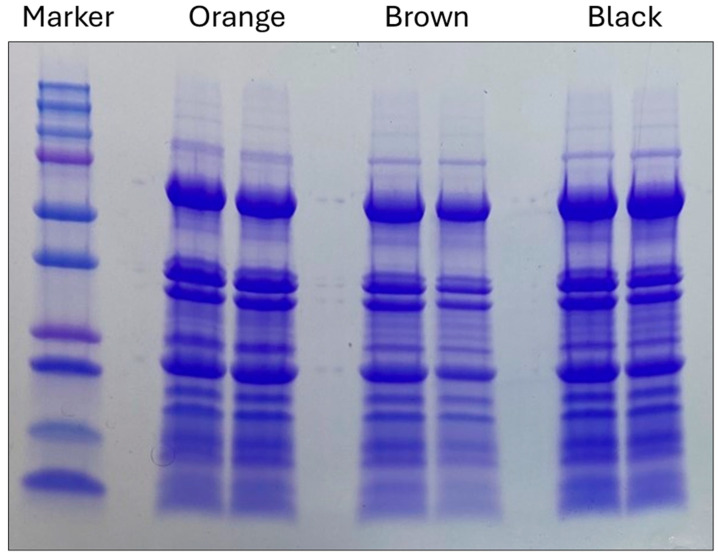
SDS PAGE (with β-mercaptoethanol, βME) comparison of the defatted extracts of pooled, pod-blasted samples that were separated into mesocarp colors (orange, brown, and black) and analyzed in duplicate. BIO-RAD™ Coomassie G-250 Stain (BIO-RAD™ Life Sciences, Hercules, CA, USA) and Any kD™ Mini-PROTEAN^®^ TGX™ with Precision Plus Dual Color Standard. Lane one shows the BIO-RAD ™ Precision Plus Protein™ Dual Color Standard ranging from 10–250 kD.

A further analysis of the protein content in each maturity class was continued by electrophoresis of two independent pooled samples, which were sorted by pod color, defatted, and then extracted with PBS buffer. Results are shown in Figure 1. SDS-PAGE, containing β-mercaptoethanol, in the comparison of defatted extracts, was separated into maturity classes and demonstrated similar composition with nearly identical banding patterns. The SSPs were the most abundant of the identified proteins in the gel and consequently may block the visualization of minor components that are differentiated from the maturity classes. Also, the dynamic changes during seed maturation may be subtle and include modifications not visible by electrophoresis. To gain a better understanding of the changes during seed maturation, a proteomics approach was introduced.

**Figure 2:**
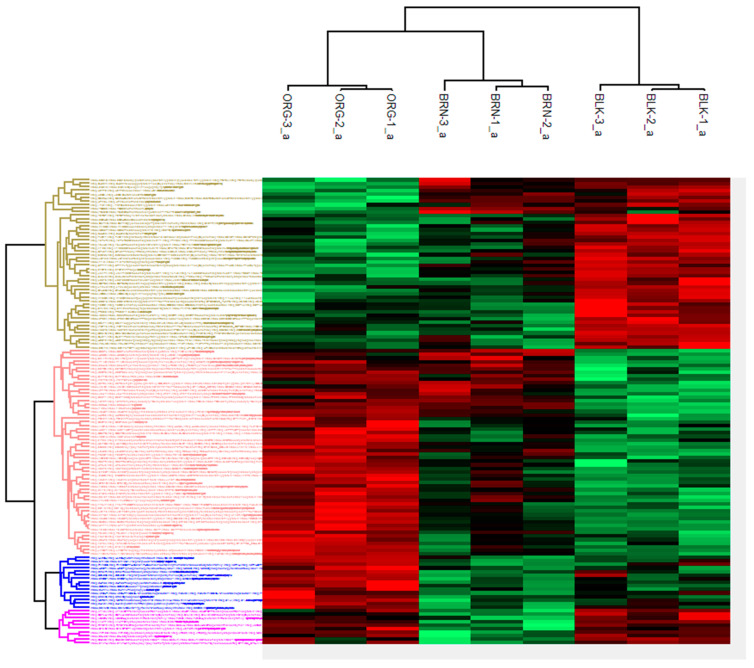
A cluster analysis of 130 proteins organized by pod color, and the relative abundance in each subcategory of maturity class. Abundance was converted to a log_2 scale and color coded according to relative amount as shown by the intensity of the red, lower concentration, and green, higher concentration, colors of the band. The names of the identified genes on the left side of the figure, is provided in Appendix A. Each category is color-coded to match the colors in this figure.

The defatted extracts of independent experiments with three replicates of pooled samples were provided by the University of Georgia research facility, and they were prepared by SDS-PAGE electrophoresis. The gel bands were excised and shipped overnight to the Proteomics Resource Center (PRC) at Rockefeller University with temperature control. Upon receipt, the protein bands were excised, treated, and extracted for further analysis by LC-MS/MS [19,20,21]. The seed extracts contained over thirteen hundred identified proteins fragmented into more than eleven thousand peptide sequences. Cluster analysis revealed 130 proteins that were differentially expressed as a function of maturity class, as shown in Figure 2. The heat map was organized to show the specific processes that were up- or down-regulated during maturation. The list of proteins identified in Figure 2 is provided in Appendix A, Table A1. The heat map provides a global picture of the temporal changes from stage to stage, but additional analysis was needed to visualize the enzymes and proteins that were different in each stage of development.

**Figure 3:**
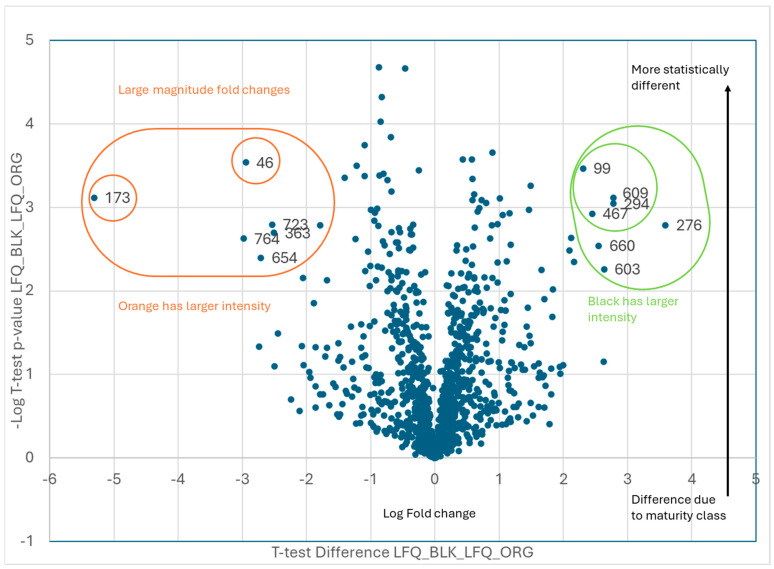
A −log10 plot of the *p* value obtained from the *t*-test vs. a log2 average intensity difference. Total proteins identified: *n* = 1047. Proteins of interest: black vs. orange maturity classes, *n* = 13.

**Figure 4:**
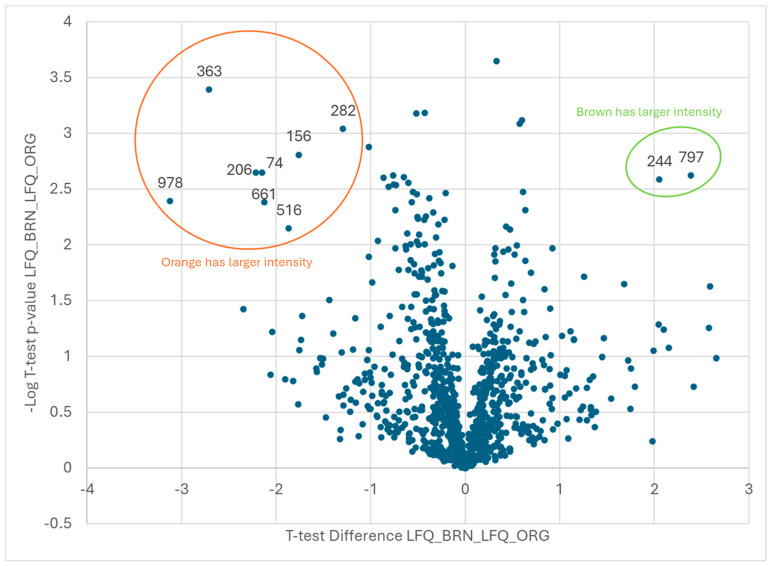
A −log10 plot of the *p* value obtained from the *t*-test vs. the log2 average intensity difference. Total proteins identified: *n* = 1047. Proteins of interest: brown vs. orange maturity classes, *n* = 10.

**Figure 5:**
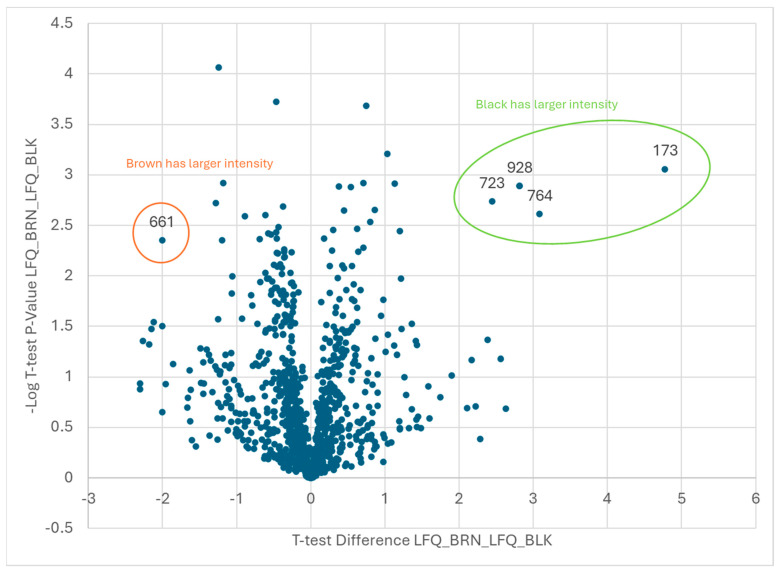
A −log10 plot of the *p* value obtained from the *t*-test vs. the log2 average intensity difference. Total proteins identified: *n* = 1047. Proteins of interest: brown vs. black maturity classes, *n* = 5.

The graphs shown in Figure 3, Figure 4 and Figure 5 represent volcano plots that visualize the differences between classes for the identification of critical expressed proteins during the final stages of maturation. An analysis of the proteomic data identified the proteins that displayed large-magnitude fold changes, as well as high statistical significance across maturity classes. We performed a 2-sample *t*-test, taking the -Log (base 10) of the *p*-values that reported the number of places after the decimal for an evaluation of the statistical significance, which is plotted on the *y*-axis. To observe practical significance, the average of the first color replicates, minus the average of the replicates for the second color, were plotted on the *x*-axis. The data points in the upper left and right corners of the volcano plots are those that were the most statistically significant with the largest magnitude fold change (FC). Plots were generated similarly for various combinations of orange/brown/black. 

Comparisons between the individual maturity classes identified 1047 proteins expressed in the seed. Further analyses focused on the proteins that were present in relatively more abundance and those of statistical significance. The comparison between the least mature kernels, orange pods, and the most mature kernels revealed 13 proteins that were differentially expressed (Figure 3, Table 1). For comparison, the ±2 Log2FC in the LFQ abundance of proteins between the proteins was used as a threshold for assessing magnitude changes. The *y*-axis shows the negative log10-scale *p*-value from the *t*-test. A minimum *p*-value of 0.05, or two places after the decimal, was used as a minimum threshold to evaluate statistical significance. 

Not surprisingly, the comparisons between the two adjacent maturity classes did not show as many up- or down-regulated proteins (Figure 4 and Figure 5) as were differentiated in the orange vs. black pod-blasted samples, which were 10 (orange/brown) and 5 (brown/black) proteins, respectively (Table 2 and Table 3). Qualitatively and anecdotally, there does appear to be a greater magnitude change when the plant matures from the orange to brown pod-blasted classes. The less mature orange pods, although not significantly different in size, performed differently when dried and roasted, thereby requiring more time to reach a desired Hunter-L color. In contrast, the brown and black pod-blasted samples performed virtually the same when dried and roasted.

## 3. Discussion

We used LC-MS-based LFQ proteomics to investigate the protein expression profile of peanut seeds that were separated into maturity classes by pod blasting to reveal the mesocarp color of the shell, which has been used as a method for assessing relative seed maturity. Previous research has reported that the macronutrient content, seed size, and seed storage protein quantity do not reveal significant differences between the last stages of pod maturation [15,17,18]. Proteomics has been used as a tool to understand the enzyme expression and regulatory mechanisms involved in the accumulation of storage reserves in crops. In peanuts, proteomic analysis has been used to characterize dynamic changes in the seed in response to disease, allergen content, and lipid accumulation in the oilseed. 

Previous studies have identified classes of proteins involved in seed maturation including nutrient accumulation, storage, and transport [5,6,7,8]. This report revealed 130 proteins that were expressed as a function of maturity classification. The functional classification of identified proteins were categorized as metabolic enzymes, nutrient accumulation/preparation/packaging, plant defense, stress response, transcriptional regulation, cell signaling, and transportation. The hierarchical clustering of the three maturity classes is shown in Figure 2. Table A1 lists all the identified proteins that were organized according to the clustering, and they are color-coded to match the heat map in Figure 2.

Additional analysis comparing each maturity subclass revealed the differentially expressed proteins that were up- or down-regulated during seed maturation (Table 1, Table 2 and Table 3). The classes of proteins include the plant defense response, metabolic enzymes, cell signaling, cell development, and acquisition of nutrients for the developing seedling upon germination.

The plant defense proteins identified include CRISP (cysteine-rich secretory proteins) and proteasome subunit Beta type-7. In plants, CRISPs are involved in immune responses to pathogens and have been shown to be part of a superfamily of secreted proteins, which are termed CAP genes [22]. Proteasomes regulate plant defense responses at several critical points, thus making this class of proteins a target for a variety of pathogens [23]. One role of the proteasome is the recycling or defense components so that the growth and development of the plant is not compromised [23]. In peanuts, there are several pervasive pathogens that are ubiquitous in all soil types and all regions including *Aspergillus flavus*, a fungus responsible for the synthesis of aflatoxins during environmental conditions such as drought and heat stress. Understanding the defense mechanisms and how pathogens interact with these critical processes are important to developing effective amelioration strategies.

The next class of differentially expressed proteins includes metabolic enzymes such as Triose Phosphate Isomerase, Pyruvate Dehydrogenase, and β-galactosidase. These proteins provide energy and carbon through the breakdown of carbohydrates and other nutrients. Storage reserves are activated during seedling growth until autotrophic growth is supported [24]. Through glycolysis, the citric acid cycle, and β-oxidation, these enzymes support the pathways that catalyze the production of ATP, thereby providing energy to the developing plant seedling.

Another category of protein classification includes the cell signaling proteins expressed in response to biotic and abiotic stress, including Heat Shock Protein 90 (HSP-90), Protein Kinases, and DEK. The reversible transfer of the y-phosphate from ATP to the amino acid side chains of proteins is catalyzed by the enzymes of the eukaryotic protein kinase superfamily. In plants, protein phosphorylation has been found to be associated with responses to various signals, such as light, hormones, stress due to temperature, nutrient deprivation, and pathogen invasion. Reversible phosphorylation also controls the activities of several metabolic and regulatory enzymes in plants [25]. As an architectural chromatin protein, DEK is linked to DNA, chromatin, and histone binding, as well as DNA-folding activities. Post-translationally, DEK can control multiple plant receptors and critical signaling nodes, which ensures that plants respond to biotic stresses in a timely and appropriate manner [26]. Plant HSP-90s are implicated in various biological processes pertaining to growth and development, as well as multiple responses to environmental stress [27]. Additionally, HSP-90 could provide a stabilization of seed storage proteins during the desiccation of the seed in preparation of dormancy [6]. Peanut seeds grow below the surface of the soil. During harvest, the plants are pulled from the soil and turned upside down on top of the ground to begin the drying process. Once the seeds reach a certain moisture level, the plants are then threshed and the pods are removed. The drying process continues until the moisture content reaches about 7% by mass. Plant mechanisms that enable the drying and stabilization of the macronutrients are critical to ensuring efficient germination, thus making this category of proteins vital to yield optimization.

Several of the identified proteins associated with cell growth and proliferation include S-Adenosyl Methionine Transferases, Phytocyanins, RNA-binding proteins, and Cell Division Cycle Proteins. RNA-binding proteins (RBPs) serve as crucial RNA regulators in the process of modulating post-transcriptional events in the cell. For instance, RBPs can identify and interact with binding motifs called RNA recognition motifs (RRMs) and/or RNA structure to develop ribonucleoprotein (RNP) complexes for the regulation of important RNA processes, including RNA stability, alternative pre-mRNA splicing, mRNA decay, translocation, post-translational nucleotide modifications, and RNA localization [28]. Phytocyanins (PCs) are a type of plant-specific blue copper proteins that serve essential roles in plant development, including the formation of nodules [29]. In the later stages of seed maturation, nodulation does not occur, so the function of this protein remains unclear. SAM (S-Adenosyl Methionine) is a central cofactor that functions as a flexible donor of the methyl group in methylation reactions that are catalyzed by several SAM-dependent methyltransferases, which direct gene expression and signaling. As seeds progress through maturation, the plant moves its resources that require the up- and down-regulation of gene expressions. Additional investigation will help to clarify the signals needed for maturity to occur, thus making them potential targets for breeding program improvements.

The last category of identified proteins include those associated with nutrient accumulation and packaging, such as glutamine synthetase, pyridoxal phosphate-dependent transferase, the acidic ribosomal protein, and the alpha glucan branching enzyme. Plant glutamine synthetase (GS, which catalyzes the synthesis of glutamine from glutamate and ammonium ions and operates as a crucial enzyme in the nitrogen metabolic pathway of organisms). Nitrogen is a critical element in the process of plant development and growth. It is also an important component in crop yield and quality formation [30]. Pyridoxal 50-phosphate (PLP), one of the active prosthetic groups of vitamin B6, is a coenzyme with unmatched catalytic versatility. It is involved in numerous biochemical reactions like transamination (transfer of amino groups), decarboxylation (removal of a carboxyl group at the β- or γ-carbon), deamination (removal of an amine group), interconversion of L and D amino acids, and racemization. PLP-dependent enzymes are primarily involved in the regulation of the biosynthesis of amino acids, amino acid-derived metabolites, amino sugars, and other amine-containing compounds [31]. Starch is an insoluble polymer of the glucose residues generated by most higher plant species and is a popular storage product of many of the seeds and storage organs produced in the agriculture industry, and it is also used for human consumption. The starch granule is an elaborate structure that includes linked glucan chains that are catalyzed by this enzyme [32]. Peanuts are an important plant-based protein providing monounsaturated fats, antioxidants, protein, and complex carbohydrates for animal and human consumption. The accumulation and packaging of macronutrients is necessary for efficient germination and nutritive quality. 

Seed storage proteins (SSPs) were found to be the most abundant proteins in the seed but did not demonstrate large magnitude fold changes in the last three maturity sub-categories. Potentially, it could be that the SSPs accumulated over all the stages significantly increased through the R5 and R6 developmental stages, as described by Li et al. [14]. The maturity classes according to mesocarp color, i.e., orange/brown/black, would most likely coincide with the R7 developmental phase in which the SSPs are packaged and prepared for dormancy and eventually germination.

## 4. Materials and Methods

### 4.1. Pod Blasting

The newly collected pods from the 2017 crop year (CY) of the same genotype as runner market-type peanuts, which were produced following typical cultural practices, were provided by the University of Georgia research facility (directed by Dr. W.D. Branch). The peanut pods were taken out of the rest of the plant and underwent “pod blasting” to uncover the mesocarp in accordance with the protocols described by Williams et al. [16]. During pod blasting, the in-shell pods were positioned in a wire basket, and their shell exterior was sprayed with high-pressure water using a residential-style pressure washer, a process that eliminates the outer layer of the peanut hull and exposes the colored mesocarp portion below. The blasted pods were subsequently split by color into three types of maturity: orange, brown, and black. Next, the rest of the pod top layer was eliminated, and the seeds were isolated for further chemical analyses. One pound of seeds from each maturity class were shipped to the Lubbock facility for further analysis.

### 4.2. Electrophoresis

Approximately 10 g of each shelled pod-blasted sample (raw, redskin) was pre-dried and defatted by blending with hexane and acetone and vacuum filtering to yield a defatted meal sample. Next, 0.1 g of the defatted peanut meal was homogenized in 1 mL of DI-H_2_O. Three replicates were pulled from the homogenized mixture. A 25 µL aliquot of the homogenized sample was mixed with 25 µL of Laemmli buffer, and 20 µL were loaded into the SDS-PAGE gel well [33]. SDS-PAGE was run using Bio-Rad Any kD(TM) Mini-PROTEAN(R) TGX(TM) Precast Gel in a Mini-PROTEAN(TM) Tetra Cell. Then, 20 uL of sample was loaded per lane. Gel was run at 150 V for 5–7 min to yield a tight, excisable single band. Running time was determined by conducting trials in collaboration with scientists at the PRC of Rockefeller University. The gel was stained in Bio-Rad Biosafe Coomassie for 1 h then destained in DI-H_2_O overnight. The band area was excised and placed in a small, sealed tube of DI-H_2_O and shipped overnight on ice packs for proteomic analysis [33].

### 4.3. Proteomic Analysis

Gel bands were excised from SDS-PAGE gel and de-stained overnight in 50 mM of Ammonium Bicarbonate/25% Acetonitrile and incubated for one hour at room temperature. The de-staining process was repeated three times. Gel bands were then dehydrated with acetonitrile following reduction with 10 mM of DTT/50 mM of Ammonium Bicarbonate for 45 min at 57 °C. After reduction, gel bands were alkylated in 45 mM of Iodoacetamide/50 mM of Ammonium Bicarbonate for 45 min at room temperature in the dark. Gel bands were dehydrated, which was followed by overnight digestion with 500 ng of Porcine Trypsin (Promega, Madison, WI, USA) and 500 ng of Endopeptidase Lys-C (Wako, Osaka, Japan) in 50 mM of Ammonium Bicarbonate. Digestion was halted by the addition of 30% acetonitrile/0.1% trifluoro acetic acid, and the peptides were extracted. Extraction was repeated twice. Samples were desalted using reversed-phase-based micro solid-phase extraction [34]. Next, 1.5 of 20 μL were injected and analyzed by nano LC-MS/MS (Q-Exactive Plus coupled to a Dionex 3000 trap-based setup, Thermo Scientific, Waltham, MA, USA). The mass spectrometer was mass calibrated weekly and operated with lock mass [35]. The MS and MS/MS were recorded at a resolution (@200 Th) of 60,000 and 30,000, respectively. The Automatic Gain Control (AGC) was set to 3 × 10^6^ and 2 × 10^5^ for the MS and MS/MS. Samples were analyzed using a 70 min gradient that increased from 2% B/98% A to 33% B/67% A in 70 min (A: 0.1% formic acid, B: 80% acetonitrile/0.1% formic acid). Peptides were separated using a 12 cm/75 um packed-in column emitter (Nikkyo Technos Co., Ltd., Tokyo, Japan).

Data were queried against the database using MaxQuant v. 1.6.0.13: Peanuts_Genemodel_17NOV2015 concatenated with common contaminants [36]. In short, a 10 ppm or 20 ppm mass accuracy was used for the precursor mass accuracy and 20 mDa for fragment ions. The carbamidomethylation of cysteines was set as a fixed modification and oxidation of methionine, and the protein N-termini acetylation was set as variable modifications. Search results were filtered using false discovery rates of 2% or better for peptides and 1% or better for proteins. Label Free Quantitation (LFQ) [37] was used to relate to the quantified matched proteins. Utilizing the Perseus software platform (v. 1.6.15.0), the LFQ values were log 2-transformed and filtered for common contaminants. The data were further filtered by requiring signals in at least two out of three replicates for at least one of the conditions, thereby resulting in 1047 proteins being quantitated. Missing values were imputed. Comparisons of the BLK vs. ORG, BRN vs. ORG, and BRN vs. BLK groups were conducted by two-sample *t*-tests (FDR-based, 0.05), as well as multiple sample ANOVA tests (FDR-based). The results of the tests were visualized using heat maps and volcano plots.

Proteins of interest were determined to be those that showed both statistical and practical significance. Statistical significance was determined using a two-tailed *t*-test for the difference between samples at *p* < 0.01. Practical significance was determined by measuring a fold change of at least 100. On Figure 3, Figure 4 and Figure 5, the proteins of interest are located in the upper corners of the graph, where −Log *p* > 2 and LogFC > 2. The volcano plots were generated by plotting the log_2-fold change (*x*-axis) against the t-test *p*-values (*y*-axis). In particular, the *y*-axis is the −log_10 (*p*-value). Consequently, if the *y*-axis is marked 1, it represents a *p*-value of 0.1. If the *y*-axis is marked 4, it represents a *p*-value of 0.0001. As a result, the data points in the upper left and right corners of the volcano plots are those that were the most statistically significant with the largest magnitude fold changes. Proteins were identified as described in the next section.

### 4.4. Identification of the Gene Name and Sequence

The gene names of the parental lines were obtained through proteomic analysis. Then, the gene name was input into the keyword search tab on the Legacy PeanutBase site at https://legacy.peanutbase.org/ (URL accessed on 30 March 2017) using the appropriate parental line. From the populated results, the gene name was selected, which showed the position of the gene on the chromosome. After the selection of the gene name, a view assembly sequence was chosen. This produced ID and sequence information. The sequence was copied and pasted into the Blast box on the Legacy PeanutBase site. Once the sequence was loaded, Tifrunner was chosen as the nucleotide database. The BLASTN button was pressed, which created search results matching the sequence of the parental line against Tifrunner. Typically, there needed to be one or two results with a high enough coverage for there to be a match, and these corresponded to the A and B genome. The locations of these sequences were input into the GBrowes Tifrunner v1 search tool on the Legacy PeanutBase website. Then, the gene name under *Arachis hypogeae* was chosen and the view assembly sequence was selected. This provided the gene ID and sequence information. 

## 5. Conclusions

In conclusion, LC-MS methods for the label-free quantification and identification of proteins in peanut seeds represent a promising technology form that can be used to examine the dynamic processes that occur during maturation. Immature seeds possess less resistance to stress; therefore, they are more prone to adverse effects during germination. Additionally, the immature seeds’ quality was lower, which affected the final product’s roasting flavor and texture. The significance of peanuts as a source of plant-based protein and heart-healthy unsaturated oil is of critical value for meeting the ongoing needs of a growing world population. Understanding the proteome during the process of plant growth and development will yield tools that can improve seed maturation and performance. 

## Figures and Tables

**Table 1:** A volcano plot comparison of the two maturity classes: orange and black pod-blasted samples. The statistically significant proteins were identified in the peanut kernels. The ±2 Log2FC in the LFQ abundance of proteins was used as a threshold for assessing the magnitude changes, and a minimum *t*-test *p*-value of 0.05 was used as a threshold to evaluate statistical significance. *n* = 13 proteins were identified as significant, and the protein identity was preliminarily determined.

Black vs. Orange	
**Orange**	
**Code**	**Protein ID**
46	Arahy|7L8B8I|Arahy.7L8B8ICAP Cysteine-richsecretoryproteins
173	Arahy|XZ8T7F|Arahy.XZ8T7Ftriosephosphateisomerase
363	Arahy|A863J5|Arahy.A863J5glutaminesynthetase2
654	Arahy|KSV57K|Arahy.KSV57Kcelldivisioncycleprotein48homolog[Glycinemax]
723	Arahy|AUI3M2|Arahy.AUI3M2Pyridoxalphosphate-dependenttransferasessuperfamilyproteinisoform
764	Arahy|SN2AIN|Arahy.SN2AINRNA-bindingprotein1
**Black**	
**Code**	**Protein ID**
99	Arahy|X0F9HV|Arahy.X0F9HVProteinkinasesuperfamilyprotein
276	Arahy|AP25S4|Arahy.AP25S460Sacidicribosomalproteinfamily
294	Arahy|TZW06C|Arahy.TZW06Cbluecopperprotein-like
467	Arahy|E04WLM|Arahy.E04WLMproteasomesubunitbetatype-7-Aprotein
603	Arahy|WK6QAZ|Arahy.WK6QAZseedstorage2Salbuminsuperfamilyprotein
609	Arahy|A9Q4ZU|Arahy.A9Q4ZUproteinDEK-likeisoformX1
660	Arahy|6Q1KS5|Arahy.6Q1KS5MethionineS-adenosyltransferase

**Table 2:** A volcano plot comparison of the two maturity classes: orange and brown pod-blasted samples. The statistically significant proteins were identified in the peanut kernels. The ±2 Log2FC in the LFQ abundance of proteins was used as a threshold for assessing magnitude changes, and a minimum *t*-test *p*-value of 0.05 was used as a threshold to evaluate statistical significance. *n* = 10 proteins were identified as significant, and the protein identity was preliminarily determined.

Brown vs. Orange	
**Orange**	
**Code**	**Protein ID**
74	Arahy|NQP1ZX|Arahy.NQP1ZX1,4-alpha-glucan-branchingenzyme-like
156	Aradu|6W1XZ|Arahy.M043T11,4-alpha-glucan-branchingenzyme-like[Glycinemax]
206	Arahy|XGW13D|Arahy.XGW13Deukaryotictranslationinitiationfactor2gammasubunit
282	Araip|GQ1CY0|Arahy.GQ1CY0beta-galactosidase17
363	Arahy|A863J5|Arahy.A863J5glutaminesynthetase2
516	Arahy|MW1RK6|Arahy.MW1RK6WDrepeat-containingprotein5-like
661	Arahy|24C55C|Arahy.24C55CpyruvatedehydrogenaseE1beta
978	Araip|T59TA|Araip.T59TAreceptor-likeproteinkinase2
**Brown**	
**Code**	**Protein ID**
244	Arahy|S6KYF2|Arahy.S6KYF2CAP(Cysteine-richsecretoryproteins
797	Arahy|EJRX2U|Arahy.EJRX2UProteinofunknownfunction(DUF1264)

**Table 3:** A volcano plot comparison of two maturity classes: brown and black pod-blasted samples. The statistically significant proteins were identified in the peanut kernels. The ±2 Log2FC in the LFQ abundance of proteins was used as a threshold for assessing magnitude changes, and a minimum *t*-test *p*-value of 0.05 was used as a threshold to evaluate statistical significance. *n* = 5 proteins were identified as significant, and the protein identity was preliminarily determined.

Brown vs. Black	
**Brown**	
**Code**	**Protein ID**
661	Arahy|24C55C|Arahy.24C55CpyruvatedehydrogenaseE1beta
**Black**	
**Code**	**Protein ID**
173	Arahy|XZ8T7F|Arahy.XZ8T7Ftriosephosphateisomerase
723	Arahy|AUI3M2|Arahy.AUI3M2Pyridoxalphosphate-dependenttransferasessuperfamilyproteinisoform
764	Arahy|SN2AIN|Arahy.SN2AINRNA-bindingprotein1
928	Arahy|L5T5KA|Arahy.L5T5KAheatshockprotein90.1

## Data Availability

The data presented in this study are available on request from the corresponding author due to privacy restrictions.

## Appendix A

**Table A1:** This table lists the proteins that were identified as up- and down-regulated during the maturation process, and it includes a cluster analysis of the 130 proteins organized by pod color and relative abundance in each subcategory of maturity class. The color-coded grouping corresponds with Figure 2 in terms of the proteins identified by sequence comparison to the diploid progenitors: *Arachis duranensis* and *Arachis ipaensis*.

Aradu|5SZ1Z|Aradu.5SZ1Zlactoylglutathionelyasefamilyprotein/glyoxalaseIfamilyprotein;Araip|PBY0V|Araip.PBY0Vlactoylglutathionelyasefamilyprotein/glyoxalaseIfamilyprotein;Aradu|T0KE9|Aradu.T0KE9nucleobase-ascorbatetransporter-likeprotein
Araip|BU32W|Araip.BU32Wbluecopperprotein-like[Glycinemax];Aradu|CI6AA|Aradu.CI6AAbluecopperprotein-like[Glycinemax]
Aradu|3N04M|Aradu.3N04MCyclophilin-likepeptidyl-prolylcis-transisomerasefamilyprotein
Araip|U2YF8|Araip.U2YF8hexokinase1;Aradu|UE1X3|Aradu.UE1X3hexokinase1
Araip|J9Q6I|Araip.J9Q6IGlutathioneS-transferasefamilyprotein
Araip|C8XNH|Araip.C8XNHGlutathioneS-transferasefamilyprotein;Aradu|EK5R9|Aradu.EK5R9GlutathioneS-transferasefamilyprotein;Araip|18DX7|Araip.18DX7glutathioneS-transferaseF3;Araip|Z2UT4|Araip.Z2UT4glutathioneS-transferasetau5;Aradu|V7D4Y|Aradu.V7D4YglutathioneS-transferaseF3;Aradu|G9I7U|Aradu.G9I7UglutathioneS-transferaseF4;Aradu|8J8HS|Aradu.8J8HSglutathioneS-transferaseF3;Araip|QEE3P|Araip.QEE3PglutathioneS-transferasetau5;Aradu|EL1FN|Aradu.EL1FNglutathioneS-transferasetau5;Araip|4CL10|Araip.4CL10glutathioneS-transferasetau5;Araip|Q3KHN|Araip.Q3KHNGlutathioneS-transferasefamilyprotein;Araip|PK7TM|Araip.PK7TMglutathioneS-transferasetau5;Araip|CV75M|Araip.CV75MglutathioneS-transferasetau5
Araip|UPW6L|Araip.UPW6Lshort-chaindehydrogenase-reductaseB
Araip|II7NF|Araip.II7NFGlutaredoxinfamilyprotein;Aradu|H2W20|Aradu.H2W20Glutaredoxinfamilyprotein
Aradu|YGS80|Aradu.YGS80nutrientreservoirprotein,putative
Aradu|YBK6Q|Aradu.YBK6QNutrientreservoir,putativen=1Tax=RicinuscommunisRepID=B9SKF4_RICCO
Araip|Y6RST|Araip.Y6RSTN-acyl-L-amino-acidamidohydrolase;Aradu|G18XJ|Aradu.G18XJN-acyl-L-amino-acidamidohydrolase
Aradu|ZQ8HD|Aradu.ZQ8HD35kDaseedmaturationprotein[Glycinemax]
Aradu|GMY4S|Aradu.GMY4SglycinecleavageT-proteinaminomethyltransferase;Araip|SA2FR|Araip.SA2FRglycinecleavageT-proteinaminomethyltransferase
Aradu|W4ZB9|Aradu.W4ZB9succinatedehydrogenase1-1;Araip|67ZY4|Araip.67ZY4succinatedehydrogenase1-1
Aradu|NS05P|Aradu.NS05Pheatshockprotein70;Araip|K60YG|Araip.K60YGheatshockprotein70
Araip|ZHH6M|Araip.ZHH6MCytochromeP450superfamilyprotein
Araip|TVG17|Araip.TVG17N-alpha-acetyltransferase15,NatAauxiliarysubunit-like[Glycinemax];Aradu|B8ABT|Aradu.B8ABTN-alpha-acetyltransferase15,NatAauxiliarysubunit-like[Glycinemax];Araip|IN65G|Araip.IN65GN-alpha-acetyltransferase15,NatAauxiliarysubunit-like[Glycinemax];Araip|9K5V6|Araip.9K5V6N-alpha-acetyltransferase15,NatAauxiliarysubunit-like[Glycinemax];Araip|WU330|Araip.WU330N-alpha-acetyltransferase15,NatAauxiliarysubunit-like[Glycinemax];Araip|UE19H|Araip.UE19HN-alpha-acetyltransferase15,NatAauxiliarysubunit-like[Glycinemax];Araip|5R0B1|Araip.5R0B1N-alpha-acetyltransferase15,NatAauxiliarysubunit-like[Glycinemax];Araip|XF9L3|Araip.XF9L3N-alpha-acetyltransferase15,NatAauxiliarysubunit-like[Glycinemax];Aradu|0NM9S|Aradu.0NM9SN-alpha-acetyltransferase15,NatAauxiliarysubunit-like[Glycinemax];Araip|2HW6R|Araip.2HW6RBTB/POZdomain-containingprotein[Glycinemax];Araip|333AM|Araip.333AMputativeMybfamilytranscriptionfactorAt1g14600-likeisoformX2[Glycinemax];Araip|XL9UX|Araip.XL9UXN-alpha-acetyltransferase15,NatAauxiliarysubunit-like[Glycinemax]
Araip|13TMR|Araip.13TMR60SribosomalproteinL27-1;Araip|YTW8M|Araip.YTW8M60SribosomalproteinL27-1;Aradu|K6L2T|Aradu.K6L2T60SribosomalproteinL27-1;Aradu|FJQ8M|Aradu.FJQ8M60SribosomalproteinL27-1;Aradu|4N88U|Aradu.4N88U60SribosomalproteinL27-1
Araip|16JUZ|Araip.16JUZRibosomalproteinS7efamilyprotein;Aradu|YQW7X|Aradu.YQW7XRibosomalproteinS7efamilyprotein
Araip|47I29|Araip.47I2960SribosomalproteinL24-2;Aradu|Q7MTE|Aradu.Q7MTE60SribosomalproteinL24-2;Araip|H35VE|Araip.H35VE60SribosomalproteinL24-2
Araip|BPV5T|Araip.BPV5TThreonyl-tRNAsynthetase;Aradu|MS8J2|Aradu.MS8J2Threonyl-tRNAsynthetase;Araip|238GJ|Araip.238GJThreonyl-tRNAsynthetase
Araip|8K0NN|Araip.8K0NNuncharacterizedproteinLOC100777508isoformX1[Glycinemax];Aradu|Y876Y|Aradu.Y876YuncharacterizedproteinLOC100777508isoformX1[Glycinemax];Araip|UA5AR|Araip.UA5ARuncharacterizedproteinLOC100777508isoformX3[Glycinemax]
Aradu|LB7M3|Aradu.LB7M3neutralalpha-glucosidase;Araip|YV6BB|Araip.YV6BBneutralalpha-glucosidase;Araip|5ND1T|Araip.5ND1Tneutralalpha-glucosidase
Aradu|77IXI|Aradu.77IXITetratricopeptiderepeat(TPR)-likesuperfamilyprotein
Araip|ZPY1F|Araip.ZPY1FL-typelectin-domaincontainingreceptorkinaseIX.1-like[Glycinemax];Aradu|P7UX8|Aradu.P7UX8L-typelectin-domaincontainingreceptorkinaseIX.1-like[Glycinemax];Aradu|WK040|Aradu.WK040L-typelectin-domaincontainingreceptorkinaseIX.1-like[Glycinemax];Aradu|K9J4G|Aradu.K9J4GL-typelectin-domaincontainingreceptorkinaseIX.1-like[Glycinemax];Araip|SHF6J|Araip.SHF6Jreceptorlectinkinase
Araip|27Q72|Araip.27Q72ATP-dependentchaperoneClpB
Araip|JM4T4|Araip.JM4T460SribosomalproteinL32-1;Araip|1L1V5|Araip.1L1V560SribosomalproteinL32-1;Aradu|YEZ8F|Aradu.YEZ8F60SribosomalproteinL32-1;Aradu|P3N99|Aradu.P3N9960SribosomalproteinL32-1
Araip|ZV5JX|Araip.ZV5JXshort-chaindehydrogenase-reductaseB;Araip|7T58U|Araip.7T58Ushort-chaindehydrogenase-reductaseB;Aradu|INB2E|Aradu.INB2Eshort-chaindehydrogenase-reductaseB;Aradu|IZX1C|Aradu.IZX1Cshort-chaindehydrogenase-reductaseB;Araip|F8GND|Araip.F8GNDshort-chaindehydrogenase-reductaseB;Aradu|W7RTE|Aradu.W7RTEshort-chaindehydrogenase-reductaseB
Araip|L6Z75|Araip.L6Z7560SribosomalL35-likeprotein;Aradu|JM0WC|Aradu.JM0WC60SribosomalL35-likeprotein
Aradu|G9PBK|Aradu.G9PBKCytosolaminopeptidasefamilyprotein;Araip|6N5UI|Araip.6N5UICytosolaminopeptidasefamilyprotein;Araip|6D01Y|Araip.6D01YCytosolaminopeptidasefamilyprotein;Aradu|G5LF8|Aradu.G5LF8Cytosolaminopeptidasefamilyprotein;Araip|7PX0B|Araip.7PX0BCytosolaminopeptidasefamilyprotein;Araip|2B3BK|Araip.2B3BKCytosolaminopeptidasefamilyprotein
Araip|L7MLK|Araip.L7MLKepoxidehydrolase;Aradu|KX25J|Aradu.KX25Jepoxidehydrolase
Araip|DAU5G|Araip.DAU5Galdo/ketoreductasefamilyoxidoreductase;Aradu|ZC28Q|Aradu.ZC28Qaldo/ketoreductasefamilyoxidoreductase;Araip|ZLU4I|Araip.ZLU4Ialdo/ketoreductasefamilyoxidoreductase;Araip|V0ZQ0|Araip.V0ZQ0aldo/ketoreductasefamilyoxidoreductase;Araip|VH5TY|Araip.VH5TYaldo/ketoreductasefamilyoxidoreductase;Araip|PLQ0G|Araip.PLQ0Galdo/ketoreductasefamilyoxidoreductase;Aradu|41CRM|Aradu.41CRMaldo/ketoreductasefamilyoxidoreductase;Araip|ZV6FQ|Araip.ZV6FQaldo/ketoreductasefamilyoxidoreductase
Aradu|VQS6V|Aradu.VQS6VCyclophilin-likepeptidyl-prolylcis-transisomerasefamilyprotein
Araip|WA3C2|Araip.WA3C2nascentpolypeptide-associatedcomplexsubunitalpha-likeprotein3;Araip|1I1JH|Araip.1I1JHnascentpolypeptide-associatedcomplexsubunitalpha-likeprotein3;Aradu|16HC5|Aradu.16HC5nascentpolypeptide-associatedcomplexsubunitalpha-likeprotein3;Aradu|44W8K|Aradu.44W8Knascentpolypeptide-associatedcomplexsubunitalpha-likeprotein3
Aradu|0ES15|Aradu.0ES15Ranbindingprotein7n=1Tax=ThalassiosirapseudonanaRepID=B8C038_THAPS;Araip|KZI9D|Araip.KZI9DRanbindingprotein7n=1Tax=ThalassiosirapseudonanaRepID=B8C038_THAPS
Araip|75DC1|Araip.75DC1cinnamoylcoareductase;Aradu|QQ3BK|Aradu.QQ3BKcinnamoylcoareductase;Araip|8BP1U|Araip.8BP1Ucinnamoylcoareductase1;Araip|34LPA|Araip.34LPAcinnamoylcoareductase;Aradu|60Z65|Aradu.60Z65NAD(P)-bindingRossmann-foldsuperfamilyprotein
Araip|8W42M|Araip.8W42Mstressup-regulatedNod19protein;Aradu|T4WFS|Aradu.T4WFSstressup-regulatedNod19protein
Araip|PRK60|Araip.PRK6017.6kDaclassIIheatshockprotein
Aradu|I095N|Aradu.I095NRibosomalproteinL6family;Araip|02QR1|Araip.02QR1RibosomalproteinL6family;Araip|7RK7R|Araip.7RK7RRibosomalproteinL6family
Aradu|KT924|Aradu.KT924DEAD-boxATP-dependentRNAhelicase-likeprotein;Aradu|6E2N9|Aradu.6E2N9DEAD-boxATP-dependentRNAhelicase-likeprotein;Araip|GL8YQ|Araip.GL8YQDEAD-boxATP-dependentRNAhelicase-likeprotein;Araip|Q0672|Araip.Q0672DEAD-boxATP-dependentRNAhelicase-likeprotein;Araip|1I9C8|Araip.1I9C8DEAD-boxATP-dependentRNAhelicase-likeprotein;Araip|TDP4I|Araip.TDP4IDEAD-boxATP-dependentRNAhelicase-likeprotein;Araip|RP4NC|Araip.RP4NCDEAD-boxATP-dependentRNAhelicase-likeprotein;Araip|SW89G|Araip.SW89GDEAD-boxATP-dependentRNAhelicase-likeprotein;Araip|4V1SV|Araip.4V1SVDEAD-boxATP-dependentRNAhelicase-likeprotein;Araip|K52G2|Araip.K52G2DEAD-boxATP-dependentRNAhelicase-likeprotein
Araip|ZBV71|Araip.ZBV71copperionbinding;Aradu|62ILE|Aradu.62ILEprobableATPsynthase24kDasubunit,mitochondrial-like[Glycinemax]
Araip|WCF75|Araip.WCF75MethionineS-adenosyltransferasen=1Tax=DetonulaconfervaceaRepID=B9ZZX3_DETCO;Aradu|WQE4S|Aradu.WQE4SMethionineS-adenosyltransferasen=1Tax=DetonulaconfervaceaRepID=B9ZZX3_DETCO;Araip|R525U|Araip.R525UMethionineS-adenosyltransferasen=1Tax=DetonulaconfervaceaRepID=B9ZZX3_DETCO;Araip|WY7N2|Araip.WY7N2MethionineS-adenosyltransferasen=1Tax=DetonulaconfervaceaRepID=B9ZZX3_DETCO;Araip|XJS45|Araip.XJS45MethionineS-adenosyltransferasen=1Tax=DetonulaconfervaceaRepID=B9ZZX3_DETCO;Aradu|X6LF1|Aradu.X6LF1MethionineS-adenosyltransferasen=1Tax=DetonulaconfervaceaRepID=B9ZZX3_DETCO;Araip|H8UEI|Araip.H8UEIMethionineS-adenosyltransferasen=1Tax=DetonulaconfervaceaRepID=B9ZZX3_DETCO;Aradu|RXW02|Aradu.RXW02MethionineS-adenosyltransferasen=1Tax=DetonulaconfervaceaRepID=B9ZZX3_DETCO
Aradu|A5ZY3|Aradu.A5ZY3mitochondrialimportreceptorsubunitTOM40-1-like[Glycinemax];Araip|9S6CK|Araip.9S6CKmitochondrialimportreceptorsubunitTOM40-1-like[Glycinemax]
Araip|D054C|Araip.D054CNADH-ubiquinoneoxidoreductase75kDasubunit;Araip|PWS7S|Araip.PWS7SNADH-ubiquinoneoxidoreductase75kDasubunit;Aradu|L20DB|Aradu.L20DBNADH-ubiquinoneoxidoreductase75kDasubunit
Araip|CL071|Araip.CL071Proteinkinasesuperfamilyprotein;Aradu|49RUG|Aradu.49RUGProteinkinasesuperfamilyprotein
Araip|H0E72|Araip.H0E72ribose-phosphatepyrophosphokinase;Aradu|72I34|Aradu.72I34ribose-phosphatepyrophosphokinase;Araip|24XA5|Araip.24XA5ribose-phosphatepyrophosphokinase;Aradu|9CV6N|Aradu.9CV6Nribose-phosphatepyrophosphokinase
Aradu|W6LXE|Aradu.W6LXEATPsynthaseF1,alphasubunit;Aradu|IY1XP|Aradu.IY1XPATPsynthaseF1,alphasubunit;Aradu|I3BW0|Aradu.I3BW0ATPsynthaseF1,alphasubunit;Aradu|G65EG|Aradu.G65EGATPsynthaseF1,alphasubunit;Aradu|BQ1D7|Aradu.BQ1D7ATPsynthaseF1,alphasubunit;Aradu|36GQX|Aradu.36GQXATPsynthaseF1,alphasubunit;Aradu|2SF5Z|Aradu.2SF5ZATPsynthaseF1,alphasubunit;Aradu|1HD8G|Aradu.1HD8GATPsynthaseF1,alphasubunit;Aradu|1AM8X|Aradu.1AM8XATPsynthaseF1,alphasubunit;Aradu|E87V5|Aradu.E87V5ATPsynthaseF1,alphasubunit;Aradu|ZR85T|Aradu.ZR85TATPsynthaseF1,alphasubunit;Aradu|RD9HF|Aradu.RD9HFATPsynthaseF1,alphasubunit;Aradu|B876B|Aradu.B876BATPsynthaseF1,alphasubunit;Aradu|AX7EV|Aradu.AX7EVATPsynthaseF1,alphasubunit;Araip|YB8JU|Araip.YB8JUATPsynthaseF1,alphasubunit
Aradu|0D47M|Aradu.0D47MTCP-1/cpn60chaperoninfamilyprotein;Araip|UP4C0|Araip.UP4C0uncharacterizedWDrepeat-containingproteinC2A9.03-likeisoformX3[Glycinemax];Aradu|55CHH|Aradu.55CHHuncharacterizedWDrepeat-containingproteinC2A9.03-likeisoformX1[Glycinemax]
Aradu|B98FL|Aradu.B98FLnutrientreservoirprotein,putative;Araip|FAG7U|Araip.FAG7Unutrientreservoirprotein,putative
Aradu|H0SGA|Aradu.H0SGAgeneralregulatoryfactor9;Araip|JB0C4|Araip.JB0C4generalregulatoryfactor9
Araip|EMI4R|Araip.EMI4Ralpha-1,4-glucan-proteinsynthase[UDP-forming]-likeprotein;Aradu|QVN0R|Aradu.QVN0Ralpha-1,4-glucan-proteinsynthase[UDP-forming]-likeprotein
Aradu|Y7IVD|Aradu.Y7IVDvacuolar-processingenzyme-like[Glycinemax];Araip|XXN6R|Araip.XXN6Rvacuolar-processingenzyme-like[Glycinemax]
Aradu|65HV5|Aradu.65HV5myo-inositoloxygenase1;Araip|LR3B6|Araip.LR3B6myo-inositoloxygenase1;Aradu|JAU6Z|Aradu.JAU6Zmyo-inositoloxygenase1
Araip|00FQ0|Araip.00FQ0Pyridoxalphosphate-dependenttransferasessuperfamilyproteinisoform1n=2Tax=TheobromacacaoRepID=UPI00042B06C0;Aradu|V4C8J|Aradu.V4C8JPyridoxalphosphate-dependenttransferasessuperfamilyproteinisoform1n=2Tax=TheobromacacaoRepID=UPI00042B06C0
Araip|K8DPE|Araip.K8DPEdolichyl-diphosphooligosaccharide--proteinglycosyltransferasesubunit1A-like[Glycinemax];Aradu|4E354|Aradu.4E354dolichyl-diphosphooligosaccharide--proteinglycosyltransferasesubunit1A-like[Glycinemax]
Araip|SVT7Q|Araip.SVT7Qisocitratelyase;Aradu|0KG04|Aradu.0KG04isocitratelyase
Araip|W9YFB|Araip.W9YFBtriosephosphateisomerase
Araip|X6PEV|Araip.X6PEVemp24/gp25L/p24family/GOLDfamilyprotein;Aradu|M0AKN|Aradu.M0AKNemp24/gp25L/p24family/GOLDfamilyprotein;Aradu|H5XDC|Aradu.H5XDCIntegralmembranecomponentofendoplasmicreticulum-derivedCOPII-coatedvesiclesn=2Tax=KomagataellapastorisRepID=C4R1A2_PICPG
Aradu|VN4G1|Aradu.VN4G140SribosomalproteinS12n=21Tax=FabaceaeRepID=I1KGU0_SOYBN;Aradu|Q0CSK|Aradu.Q0CSK40SribosomalproteinS12n=21Tax=FabaceaeRepID=I1KGU0_SOYBN;Araip|XI5DK|Araip.XI5DK40SribosomalproteinS12n=21Tax=FabaceaeRepID=I1KGU0_SOYBN;Araip|MS30Q|Araip.MS30Q40SribosomalproteinS12n=21Tax=FabaceaeRepID=I1KGU0_SOYBN;Araip|RI7AH|Araip.RI7AH40SribosomalproteinS12n=21Tax=FabaceaeRepID=I1KGU0_SOYBN;Aradu|T2SCC|Aradu.T2SCC40SribosomalproteinS12n=2Tax=PapilionoideaeRepID=I1KVK9_SOYBN;Aradu|NWM4M|Aradu.NWM4M40SribosomalproteinS12n=2Tax=PapilionoideaeRepID=I1KVK9_SOYBN
Aradu|KP7PE|Aradu.KP7PEuricase-2isozyme2[Glycinemax];Araip|UM9DW|Araip.UM9DWuricase-2isozyme2[Glycinemax];Aradu|C7VL7|Aradu.C7VL7CHD3-typechromatin-remodelingfactorpickleprotein
Aradu|J5HIY|Aradu.J5HIYmalatedehydrogenase;Araip|KT3YI|Araip.KT3YImalatedehydrogenase;Aradu|N8RFP|Aradu.N8RFPmalatedehydrogenase
Araip|B69F1|Araip.B69F126Sproteasomenon-ATPaseregulatorysubunit-likeprotein;Aradu|RT222|Aradu.RT22226Sproteasomenon-ATPaseregulatorysubunit-likeprotein;Aradu|L82NR|Aradu.L82NR26Sproteasomenon-ATPaseregulatorysubunit-likeprotein;Araip|9S7LJ|Araip.9S7LJ26Sproteasomenon-ATPaseregulatorysubunit-likeprotein;Aradu|QC0XQ|Aradu.QC0XQ26Sproteasomenon-ATPaseregulatorysubunit-likeprotein;Aradu|623R5|Aradu.623R526Sproteasomenon-ATPaseregulatorysubunit-likeprotein
Araip|1ZF2P|Araip.1ZF2P3-hydroxyisobutyryl-CoAhydrolase-likeprotein;Aradu|TQ8V3|Aradu.TQ8V33-hydroxyisobutyryl-CoAhydrolase-likeprotein;Aradu|65YWZ|Aradu.65YWZ3-hydroxyisobutyryl-CoAhydrolase-likeprotein3,mitochondrial-likeisoform1[Glycinemax]
Aradu|88EH5|Aradu.88EH5kunitztrypsininhibitor1
Aradu|7I20U|Aradu.7I20Utriosephosphateisomerase
Aradu|V8HSY|Aradu.V8HSY3-isopropylmalatedehydratase,smallsubunit;Araip|LP8AE|Araip.LP8AE3-isopropylmalatedehydratase,smallsubunit
Araip|UHR96|Araip.UHR96staphylococcalnucleasedomain-containingprotein1-like[Glycinemax];Aradu|335WD|Aradu.335WDstaphylococcalnucleasedomain-containingprotein1-like[Glycinemax]
Araip|PQA2W|Araip.PQA2WPhosphopyruvatehydratasen=1Tax=Dictyosteliumfasciculatum(strainSH3)RepID=F4PJ27_DICFS;Aradu|07WQA|Aradu.07WQAPhosphopyruvatehydratasen=1Tax=Dictyosteliumfasciculatum(strainSH3)RepID=F4PJ27_DICFS
Araip|C8DTK|Araip.C8DTKubiquitinactivatingenzyme2
Aradu|4AQ1Z|Aradu.4AQ1Zadenylosuccinatesynthetase;Araip|HUN8L|Araip.HUN8Ladenylosuccinatesynthetase;Araip|YLK1G|Araip.YLK1Gadenylosuccinatesynthetase;Aradu|RX3FZ|Aradu.RX3FZadenylosuccinatesynthetase
Aradu|UKZ71|Aradu.UKZ71UDP-glucuronicaciddecarboxylase6-like[Glycinemax];Araip|V5PPP|Araip.V5PPPUDP-glucuronicaciddecarboxylase6-like[Glycinemax];Araip|QUX0P|Araip.QUX0PUDP-glucuronicaciddecarboxylase6-like[Glycinemax];Aradu|X23MZ|Aradu.X23MZUDP-glucuronicaciddecarboxylase6-like[Glycinemax];Araip|I2Y83|Araip.I2Y83UDP-glucuronicaciddecarboxylase5[Glycinemax];Aradu|42MBK|Aradu.42MBKUDP-glucuronicaciddecarboxylase5-likeisoformX3[Glycinemax];Araip|Y9HHS|Araip.Y9HHSUDP-glucuronicaciddecarboxylase1;Araip|UGL36|Araip.UGL36UDP-glucuronicaciddecarboxylase1;Aradu|W3UAF|Aradu.W3UAFUDP-glucuronicaciddecarboxylase1;Aradu|F3XNS|Aradu.F3XNSUDP-glucuronicaciddecarboxylase1;Aradu|G5902|Aradu.G5902UDP-glucuronicaciddecarboxylase1;Aradu|BAA8F|Aradu.BAA8FUDP-glucuronicaciddecarboxylase1;Araip|S12BL|Araip.S12BLUDP-glucuronicaciddecarboxylase1
Araip|GD3PG|Araip.GD3PGheatshockprotein70;Aradu|DLG8U|Aradu.DLG8Uheatshockprotein70;Araip|4W6ZL|Araip.4W6ZLheatshockprotein70;Araip|6D8Q6|Araip.6D8Q6heatshockprotein70;Aradu|2IN3E|Aradu.2IN3Eheatshockprotein70;Aradu|L2DZ2|Aradu.L2DZ2heatshockprotein70;Aradu|TET8U|Aradu.TET8Uheatshockprotein70;Aradu|0J1NB|Aradu.0J1NBheatshockprotein70;Araip|SDN9Y|Araip.SDN9Yheatshockprotein70;Aradu|XG1KZ|Aradu.XG1KZheatshockprotein70;Araip|NU4GY|Araip.NU4GYheatshockprotein70
Aradu|AZC7U|Aradu.AZC7Unucleartransportfactor2B
Araip|2IF8M|Araip.2IF8Meukaryotictranslationinitiationfactor2gammasubunit;Aradu|92DR8|Aradu.92DR8eukaryotictranslationinitiationfactor2gammasubunit;Aradu|Q8YUB|Aradu.Q8YUBeukaryotictranslationinitiationfactor2gammasubunit;Araip|Z4ZAK|Araip.Z4ZAKeukaryotictranslationinitiationfactor2gammasubunit
Araip|H6WCB|Araip.H6WCBperoxisomal(S)-2-hydroxy-acidoxidaseGLO1;Araip|S6Q95|Araip.S6Q95peroxisomal(S)-2-hydroxy-acidoxidaseGLO1;Aradu|U8IBL|Aradu.U8IBLperoxisomal(S)-2-hydroxy-acidoxidaseGLO1
Araip|6S389|Araip.6S389Acyl-[acyl-carrier-protein]desaturasen=2Tax=SolanumRepID=K4C635_SOLLC;Aradu|L1M1M|Aradu.L1M1MAcyl-[acyl-carrier-protein]desaturasen=2Tax=SolanumRepID=K4C635_SOLLC
Araip|L8U0E|Araip.L8U0EmalonylCoA-acylcarriertransacylase;Araip|R08HU|Araip.R08HUmalonylCoA-acylcarriertransacylase;Aradu|AR6IT|Aradu.AR6ITmalonylCoA-acylcarriertransacylase;Aradu|2W51Q|Aradu.2W51QmalonylCoA-acylcarriertransacylase;Araip|TR8WR|Araip.TR8WRmalonylCoA-acylcarriertransacylase
Araip|F83CP|Araip.F83CPPyruvatekinasefamilyprotein;Aradu|P81AE|Aradu.P81AEPyruvatekinasefamilyprotein
Aradu|4Q29Q|Aradu.4Q29QphospholipaseDP2;Aradu|FS7LG|Aradu.FS7LGphospholipaseDP2;Araip|0C2UG|Araip.0C2UGphospholipaseDalpha1;Aradu|H7I4I|Aradu.H7I4IphospholipaseDalpha1
Araip|R86PR|Araip.R86PRNAD-dependentepimerase/dehydratasefamilyprotein;Aradu|ZL6EF|Aradu.ZL6EFNAD-dependentepimerase/dehydratasefamilyprotein;Araip|N3G5W|Araip.N3G5WNAD-dependentepimerase/dehydratasen=7Tax=MethylobacteriumRepID=A9VXU6_METEP;Araip|U179F|Araip.U179FNAD-dependentepimerase/dehydratasen=7Tax=MethylobacteriumRepID=A9VXU6_METEP
Araip|UF6J5|Araip.UF6J5serinehydroxymethyltransferase4;Aradu|BAW60|Aradu.BAW60serinehydroxymethyltransferase4
Araip|A03F3|Araip.A03F3aspartateaminotransferase5;Aradu|GKD3R|Aradu.GKD3Raspartateaminotransferase5
Aradu|9W6CT|Aradu.9W6CTglucose-6-phosphatedehydrogenase6;Aradu|G9N9R|Aradu.G9N9Rglucose-6-phosphatedehydrogenase6;Araip|52S9A|Araip.52S9Aglucose-6-phosphatedehydrogenase5;Araip|Z8HBD|Araip.Z8HBDglucose-6-phosphatedehydrogenase6;Araip|8D8E0|Araip.8D8E0glucose-6-phosphatedehydrogenase6;Araip|L2MWP|Araip.L2MWPglucose-6-phosphatedehydrogenase6;Araip|Y3H9F|Araip.Y3H9Fglucose-6-phosphatedehydrogenase6
Araip|I6NSD|Araip.I6NSDtransaldolasefamilyprotein;Aradu|U0DT9|Aradu.U0DT9transaldolasefamilyprotein;Aradu|472P6|Aradu.472P6transaldolasefamilyprotein;Aradu|NKW3P|Aradu.NKW3Preceptorkinase2
Araip|4XU2W|Araip.4XU2Welongationfactor1-beta;Aradu|JV10F|Aradu.JV10Felongationfactor1-beta
Aradu|GP04Q|Aradu.GP04Qpyruvatedecarboxylase-2;Araip|8UW4J|Araip.8UW4Jpyruvatedecarboxylase-2
Aradu|7K065|Aradu.7K065malatedehydrogenase;Aradu|5S6XB|Aradu.5S6XBmalatedehydrogenase;Araip|Q2R8P|Araip.Q2R8Pmalatedehydrogenase
Araip|KVI16|Araip.KVI16acetyl-CoAcarboxylase2;Aradu|ET2TE|Aradu.ET2TEacetyl-CoAcarboxylase2
Aradu|8IN6N|Aradu.8IN6NPyruvatekinasefamilyprotein;Araip|L7NBZ|Araip.L7NBZPyruvatekinasefamilyprotein
Aradu|9645F|Aradu.9645Fproteindisulfideisomerase-related;Araip|7X1IR|Araip.7X1IRproteindisulfideisomerase-likeprotein;Aradu|IJE78|Aradu.IJE78proteindisulfideisomerase-likeprotein;Aradu|L73E9|Aradu.L73E9proteindisulfideisomerase-likeprotein;Araip|34ZNV|Araip.34ZNVprobableproteindisulfide-isomeraseA6-likeisoform1[Glycinemax]
Aradu|7WJ9D|Aradu.7WJ9DSec14p-likephosphatidylinositoltransferfamilyprotein
Araip|776JT|Araip.776JTRABGDPdissociationinhibitor2;Aradu|RCS61|Aradu.RCS61RABGDPdissociationinhibitor2;Araip|FU5J9|Araip.FU5J9RABGDPdissociationinhibitor2;Aradu|SJR7G|Aradu.SJR7GRABGDPdissociationinhibitor2;Araip|36SP7|Araip.36SP7RABGDPdissociationinhibitor2;Aradu|PD7X7|Aradu.PD7X7RABGDPdissociationinhibitor2
Araip|0B1IX|Araip.0B1IXpyruvatedehydrogenaseE1component,alphasubunit;Aradu|PJ5MX|Aradu.PJ5MXpyruvatedehydrogenaseE1component,alphasubunit
Aradu|JB1F3|Aradu.JB1F3Dihydrolipoamideacetyltransferasecomponent(E2)ofpyruvatedehydrogenasecomplexn=7Tax=BacteriaRepID=F7URM9_SYNYG;Aradu|RRB5Y|Aradu.RRB5Ydihydrolipoyllysine-residueacetyltransferasecomponentofpyruvatedehydrogenasecomplex,mitochondrial-likeisoformX1[Glycinemax]
Aradu|M7N57|Aradu.M7N57staphylococcalnucleasedomain-containingprotein1-like[Glycinemax];Araip|U14XR|Araip.U14XRstaphylococcalnucleasedomain-containingprotein1-like[Glycinemax]
Aradu|49UB9|Aradu.49UB9Pyruvatekinasefamilyprotein;Araip|GCV0S|Araip.GCV0SPyruvatekinasefamilyprotein;Aradu|YED6D|Aradu.YED6DPentatricopeptiderepeat(PPR-like)superfamilyprotein;Araip|WH1S2|Araip.WH1S2Pentatricopeptiderepeat(PPR-like)superfamilyprotein
Aradu|C5DXV|Aradu.C5DXVfructose-bisphosphatealdolase2;Araip|ZR190|Araip.ZR190fructose-bisphosphatealdolase2;Araip|07C53|Araip.07C53fructose-bisphosphatealdolase2;Araip|60WE7|Araip.60WE7fructose-bisphosphatealdolase2;Aradu|Z5F9U|Aradu.Z5F9Ufructose-bisphosphatealdolase1;Aradu|N8WG9|Aradu.N8WG9fructose-bisphosphatealdolase2;Araip|GJ91G|Araip.GJ91Gfructose-bisphosphatealdolase1
Aradu|HW77V|Aradu.HW77Vglutathionereductase,cytosolic-likeisoformX3[Glycinemax]
Aradu|19IZS|Aradu.19IZSTransketolase;Aradu|IS5YT|Aradu.IS5YTTransketolase;Araip|QYZ6U|Araip.QYZ6UTransketolase
Araip|T0LEM|Araip.T0LEM1,4-alpha-glucan-branchingenzyme-like[Glycinemax]
Araip|7XU1G|Araip.7XU1GInositolmonophosphatasefamilyprotein
Aradu|K9HT3|Aradu.K9HT3glycogen/starch/alpha-glucanphosphorylasefamilyprotein;Aradu|EMA8S|Aradu.EMA8Sglycogen/starch/alpha-glucanphosphorylasefamilyprotein;Araip|NE7CK|Araip.NE7CKglycogen/starch/alpha-glucanphosphorylasefamilyprotein
Araip|15F3V|Araip.15F3VCalreticulin2,calcium-bindingproteinn=1Tax=CoccomyxasubellipsoideaC-169RepID=I0YTB6_9CHLO;Aradu|59RNH|Aradu.59RNHCalreticulin2,calcium-bindingproteinn=1Tax=CoccomyxasubellipsoideaC-169RepID=I0YTB6_9CHLO
Araip|91947|Araip.91947glutaminesynthetase2
Araip|LWG2P|Araip.LWG2P3-isopropylmalatedehydratase,largesubunit;Aradu|2Q562|Aradu.2Q5623-isopropylmalatedehydratase,largesubunit
Aradu|14CMN|Aradu.14CMNacetyl-CoAcarboxylase,carboxyltransferase,alphasubunit;Araip|7H2NS|Araip.7H2NSacetyl-CoAcarboxylase,carboxyltransferase,alphasubunit
Araip|UL2GU|Araip.UL2GUglutaminesynthetase2;Aradu|G6IK8|Aradu.G6IK8glutaminesynthetase2
Aradu|6W1XZ|Aradu.6W1XZ1,4-alpha-glucan-branchingenzyme-like[Glycinemax]
Araip|R1WSG|Araip.R1WSGSPFH/Band7/PHBdomain-containingmembrane-associatedproteinfamily;Araip|AKR1H|Araip.AKR1HSPFH/Band7/PHBdomain-containingmembrane-associatedproteinfamily;Aradu|G1SYE|Aradu.G1SYESPFH/Band7/PHBdomain-containingmembrane-associatedproteinfamily;Aradu|0B0KP|Aradu.0B0KPSPFH/Band7/PHBdomain-containingmembrane-associatedproteinfamily;Aradu|F950X|Aradu.F950XSPFH/Band7/PHBdomain-containingmembrane-associatedproteinfamily;Araip|NDR0B|Araip.NDR0BSPFH/Band7/PHBdomain-containingmembrane-associatedproteinfamily;Araip|CE7Q8|Araip.CE7Q8SPFH/Band7/PHBdomain-containingmembrane-associatedproteinfamily;Araip|M9Q5C|Araip.M9Q5CSPFH/Band7/PHBdomain-containingmembrane-associatedproteinfamily;Aradu|M7QCJ|Aradu.M7QCJSPFH/Band7/PHBdomain-containingmembrane-associatedproteinfamily
Aradu|H2SP1|Aradu.H2SP1phosphoenolpyruvatecarboxylase1;Araip|NKX5E|Araip.NKX5Ephosphoenolpyruvatecarboxylase1;Araip|C5W32|Araip.C5W32phosphoenolpyruvatecarboxylase2;Araip|6YH6X|Araip.6YH6Xphosphoenolpyruvatecarboxylase2;Aradu|1TH42|Aradu.1TH42phosphoenolpyruvatecarboxylase1;Aradu|3579H|Aradu.3579Hglucose-6-phosphateisomerase;Aradu|IW635|Aradu.IW635phosphoenolpyruvatecarboxylase1;Aradu|UP5AZ|Aradu.UP5AZphosphoenolpyruvatecarboxylase1;Araip|C3PGN|Araip.C3PGNphosphoenolpyruvatecarboxylase1;Aradu|P6TAV|Aradu.P6TAVphosphoenolpyruvatecarboxylase3
Araip|02N4R|Araip.02N4Risocitratedehydrogenase;Aradu|452B2|Aradu.452B2isocitratedehydrogenase
Araip|EDV8G|Araip.EDV8G1,4-alpha-glucan-branchingenzyme-like[Glycinemax];Aradu|37TWH|Aradu.37TWHstarchbranchingenzymeI
Aradu|82DSF|Aradu.82DSFcytokininoxidase/dehydrogenase1;Araip|ZXC56|Araip.ZXC56cytokininoxidase/dehydrogenase1
Araip|KHF35|Araip.KHF35Phosphoglyceratemutase,2,3-bisphosphoglycerate-independent
Aradu|BD60N|Aradu.BD60NGlucose-1-phosphateadenylyltransferasefamilyprotein
Aradu|0UW7J|Aradu.0UW7JPhosphoglyceratekinasefamilyprotein
Aradu|V2CUT|Aradu.V2CUTFASCICLIN-likearabinogalactan1;Araip|WZM84|Araip.WZM84FASCICLIN-likearabinogalactan1;Araip|F7QJH|Araip.F7QJHFASCICLIN-likearabinogalactan1;Aradu|83VKU|Aradu.83VKUFASCICLIN-likearabinogalactan1
Araip|32X4C|Araip.32X4Cphosphoenolpyruvatecarboxylase1
Araip|N8RKW|Araip.N8RKWheatshockprotein70;Aradu|24A4H|Aradu.24A4Hheatshockprotein70;Araip|Z1IJI|Araip.Z1IJIheatshockprotein70;Araip|S73ZI|Araip.S73ZIheatshockprotein70
Araip|3H4X4|Araip.3H4X4glutamine-tRNAligase,putative/glutaminyl-tRNAsynthetase,putative/GlnRS,putative
Aradu|094MD|Aradu.094MDdelta-1-pyrroline-5-carboxylatesynthetase;Araip|89D00|Araip.89D00delta-1-pyrroline-5-carboxylatesynthetase
Araip|V71XV|Araip.V71XVGTP-bindingnuclearRan-likeprotein;Aradu|V9UDT|Aradu.V9UDTGTP-bindingnuclearRan-likeprotein;Araip|P6YY9|Araip.P6YY9GTP-bindingnuclearRan-likeprotein;Aradu|03JH0|Aradu.03JH0GTP-bindingnuclearRan-likeprotein
Araip|G0FLH|Araip.G0FLH40SribosomalproteinS13[Glycinemax];Araip|CAL49|Araip.CAL4940SribosomalproteinS13[Glycinemax];Aradu|79MUY|Aradu.79MUY40SribosomalproteinS13[Glycinemax];Aradu|U6Y5X|Aradu.U6Y5XPentatricopeptiderepeat(PPR)superfamilyprotein;Aradu|NIR3G|Aradu.NIR3G40SribosomalproteinS13[Glycinemax];Araip|JK8VR|Araip.JK8VR40SribosomalproteinS13[Glycinemax]
Araip|VS9DN|Araip.VS9DNHyaluronan/mRNAbindingfamily;Aradu|FK6GI|Aradu.FK6GIHyaluronan/mRNAbindingfamily;Araip|FGD4N|Araip.FGD4NHyaluronan/mRNAbindingfamily
Araip|44UYF|Araip.44UYFDihydroxyacetonekinase;Aradu|EI99W|Aradu.EI99WDihydroxyacetonekinase
Araip|RWB72|Araip.RWB72NAD-dependentmalicenzyme1;Aradu|S56JH|Aradu.S56JHNAD-dependentmalicenzyme1;Araip|J5GX8|Araip.J5GX8NAD-dependentmalicenzyme1;Aradu|S5K3Z|Aradu.S5K3ZNAD-dependentmalicenzyme1;Araip|JZ063|Araip.JZ063NAD-dependentmalicenzyme1;Aradu|0C9TU|Aradu.0C9TUNAD-dependentmalicenzyme1;Aradu|CK0W6|Aradu.CK0W6NAD-dependentmalicenzyme2;Araip|F77JF|Araip.F77JFNAD-dependentmalicenzyme2;Aradu|P6639|Aradu.P6639NAD-dependentmalicenzyme1;Aradu|R9UNN|Aradu.R9UNNNADP-malicenzyme1;Araip|F5MI5|Araip.F5MI5NADP-malicenzyme4;Araip|G1WPG|Araip.G1WPGNAD-dependentmalicenzyme2
Aradu|C73IQ|Aradu.C73IQTCP-1/cpn60chaperoninfamilyprotein;Araip|J0WZR|Araip.J0WZRTCP-1/cpn60chaperoninfamilyprotein
Aradu|WR10B|Aradu.WR10BpyruvatedehydrogenaseE1beta;Araip|VR692|Araip.VR692pyruvatedehydrogenaseE1beta;Araip|NEM0P|Araip.NEM0PpyruvatedehydrogenaseE1beta;Aradu|N2G7A|Aradu.N2G7ApyruvatedehydrogenaseE1beta
Aradu|BNJ3E|Aradu.BNJ3Eprobableaspartylaminopeptidase-like[Glycinemax]
Araip|GN6XE|Araip.GN6XE40SribosomalproteinS3a-1;Aradu|S59LW|Aradu.S59LW40SribosomalproteinS3a-1;Araip|CH8XD|Araip.CH8XD40SribosomalproteinS3a-1
Aradu|07VYH|Aradu.07VYH3-hydroxyacyl-[acyl-carrier-protein]dehydrataseFabZn=2Tax=SynechococcusRepID=FABZ_SYNJA;Araip|5MC2N|Araip.5MC2N3-hydroxyacyl-[acyl-carrier-protein]dehydrataseFabZn=2Tax=SynechococcusRepID=FABZ_SYNJA
